# Delving Into the Dialogue between Epigenetic Modifications and Immunometabolism in Cancer

**DOI:** 10.1002/ggn2.202500034

**Published:** 2025-09-05

**Authors:** Xiaowen Xie, Weici Liu, Pengpeng Zhang, Peng Luo, Bufu Tang, Wenjun Mao

**Affiliations:** ^1^ Department of Thoracic Surgery the Affiliated Wuxi People's Hospital of Nanjing Medical University, Wuxi People's Hospital Wuxi Medical Center Nanjing Medical University Wuxi 214023 China; ^2^ Department of Lung Cancer Tianjin Lung Cancer Center National Clinical Research Center for Cancer Key Laboratory of Cancer Prevention and Therapy Tianjin's Clinical Research Center for Cancer Tianjin Medical University Cancer Institute and Hospital Tianjin 300060 China; ^3^ Department of Oncology Zhujiang Hospital Southern Medical University Guangzhou 510282 China; ^4^ Department of Interventional Radiology Zhongshan Hospital Shanghai Institute of Medical Imaging National Clinical Research Center of Interventional Medicine Fudan University Shanghai 200032 China

**Keywords:** epigenetic modification, metabolic reprogramming, single‐cell sequencing, spatial omics, tumor immunometabolism

## Abstract

Tumor immunometabolism and epigenetic modifications are intricately linked in reshaping the tumor microenvironment, with their crosstalk offering novel insights into cancer biology. Nutrient deprivation and metabolic byproducts drive metabolic reprogramming in immune cells, where metabolites act as epigenetic modulators to regulate immune‐related gene expression and influence immune cell activation, differentiation, and functional states. The cellular complexity, dynamic interactions, and spatiotemporal heterogeneity of the tumor microenvironment pose significant challenges to current studies. Emerging technologies such as single‐cell sequencing, spatial omics, and artificial intelligence provide powerful tools to address these complexities. This perspective discusses the crosstalk between tumor immunometabolism and epigenetic modifications, while also exploring how the emerging technologies may advance mechanistic insights and therapeutic innovation in this field.

## Graphical abstract

Epigenetic modifications—including DNA, histone, and RNA modifications—and their interplay play pivotal roles in tumorigenesis and progression. The crosstalk between tumor immunometabolism and epigenetic regulation has garnered significant attention, as metabolites serve as epigenetic modulators to modulate immune cell activation, differentiation, and function. Despite the complexity of the tumor microenvironment, emerging technologies like single‐cell sequencing, spatial omics, and artificial intelligence are driving breakthroughs in this field.

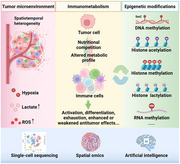

## 1 Introduction

Tumor cells exhibit a distinct metabolic phenotype, reprogramming their glucose, lipid, and amino acid metabolism through multiple signaling pathways, with aerobic glycolysis (the Warburg effect) as the most prominent metabolic feature. This metabolic adaptation not only fulfills the high energy demands of tumor cells but also modulates the function and activity of immune cells in the tumor microenvironment (TME).^[^ ^1^ ^]^ The acidic, hypoxic, and nutrient‐deprived TME drives metabolic reprogramming in immune cells, enabling them to sustain proliferation, differentiation, and functions. Mechanistically, metabolites and metabolic enzymes act as pivotal immunoregulatory mediators, integrating energy metabolism with signaling cascades to modulate immune cell activity, thereby critically shaping immunotherapy efficacy.^[^ ^2^ ^]^

Epigenetics is defined as “the study of molecules and mechanisms that can perpetuate alternative gene activity states in the context of the same DNA sequence”.^[^ ^3^ ^]^ Aberrant epigenetic modifications—including DNA, histone, and RNA modifications—have been demonstrated to play pivotal roles in tumorigenesis and progression, serving as a common molecular hallmark of tumors.^[^ ^4^ ^]^ Investigating the crosstalk between epigenetic modifications and tumor immunometabolism provides novel insights into cancer biology. In previous work, we systematically summarized the characteristics of tumor‐associated epigenomic modifications and their regulatory roles in tumor metabolic reprogramming and immunometabolism.^[^ ^5^ ^]^ Herein, this perspective further explores the interplay between epigenetic modifications—including RNA modifications—and tumor immunometabolism, along with the emerging technologies that facilitate their investigation.

## 2 Common Epigenetic Modifications Modulating Tumor Immunity

Epigenetic modifications serve as a critical nexus bridging genetics, environment, and disease, playing pivotal roles in diverse physiological processes and disease progression. These modifications are heritable yet reversible, which are dynamically regulated by “writers”, “readers”, and “erasers” (**Table** **1**).^[^ ^3^ ^]^ Dysregulated epigenetic modifications, driven by multiple factors, promote tumor initiation and progression by altering the expression of tumor‐associated genes and modulating crucial cellular processes.

**Table 1:** Summary of common epigenetic modifications.

Modification type		Writer	Eraser	Reader
DNA modification	DNA methylation	DNMTs	TETs	MBD proteins, UHRF1/2, zinc‐finger proteins
Histone modification	Histone acetylation	HATs (e.g., p300/CBP, GCN5)	HDACs, SIRTs	Bromodomains, DPF, and YEATS domain‐containing proteins
	Histone methylation	HMTs (e.g., EZH2, SETD1A)	HDMs (e.g., KDM6A, LSD1)	MBT domain‐containing proteins, chromo domain‐containing proteins, PHD domain‐containing proteins
	Histone phosphorylation	protein kinases (e.g., Aurora B kinase, JAK kinase)	protein phosphatases (e.g., protein phosphatase 1)	BRCT domain‐containing proteins, 14‐3‐3 proteins
	Histone ubiquitination	E3 Ligases (e.g., RING1B/BMI1)	deubiquitinating enzymes (e.g., USP22)	PRC1/2, DNMT1, GCN5
	Histone lactylation	p300, GCN5, HBO1	HDAC1/2/3, SIRT1/3	Brg1
RNA modification	RNA methylation	METTL3/METTL14 complex	FTO, ALKBH5	YTHDF1/2/3, YTHDC1/2

Abbreviations: ALKBH5, alkB homologue 5; BMI1, B‐cell‐specific Moloney murine leukemia virus insertion site 1; BRCT domain, BRCA1 C‐terminal domain; Brg1, brahma‐related gene 1; DNMTs, DNA methyltransferases; DPF, double PHD finger; EZH2, enhancer of zeste homolog 2; FTO, fat mass and obesity‐associated protein; GCN5, general control non‐depressible 5; HATs, histone acetyltransferases; HBO1, KAT7; HDACs, histone deacetylases; HDMs, histone demethylases; HMTs, histone methyltransferase; JAK, Janus kinase; KDM6A, lysine specific demethylase 6A; LSD1, lysine specific demethylase 1; MBD, methyl‐CpG‐binding‐domain; MBT, malignant brain tumor; METTL14, methyltransferase‐like 14; METTL3, methyltransferase‐like 3; p300/CBP, E1A‐binding protein/CREB‐binding protein; PHD, plant homeodomain; PRC1/2, polycomb repressive complex 1/2; RING1B, ring finger protein 1B; SETD1A, SET domain containing 1A; SIRTs, Sirtuins; TETs, ten‐eleven translocation family proteins; UHRF1/2, ubiquitin‐like with PHD and RING finger domains 1/2; USP22, ubiquitin specific peptidase 22; YTHDF1/2/3, YTH N6‐methyladenosine RNA binding protein F1/2/3; YTHDC1/2, YTH N6‐methyladenosine RNA binding protein C1/2.

Aberrant epigenetic modifications drive tumor proliferation and metastasis by regulating gene expression and immune responses. For instance, decitabine‐mediated DNA methylation inhibition reactivates silenced antitumor immune genes in advanced tumors, thereby augmenting tumor‐infiltrating lymphocytes and attenuating tumor progression.^[^ ^6^ ^]^ Interleukin‐9 (IL‐9) secretion from leukemia stem cells activates Janus kinase (JAK)‐signal transduction and activator of transcription (STAT) signaling in CD4⁺ T cells, enhancing histone 3 lysine 4 methylation (H3K4me). This induces type 1 helper T cell polarization and the release of interferon‐gamma (IFN‐γ) and tumor necrosis factor‐alpha (TNF‐α), which in turn drive leukemia stem cell expansion in a positive feedback loop.^[^ ^7^ ^]^ Furthermore, histone lactylation has emerged as a novel epigenetic modification bridging cellular metabolism and epigenetic regulation, attracting increasing attention. A recent study revealed that acetyl coenzyme A (acetyl‐CoA) synthetase 2 (ACSS2) and lysine acetyltransferase 2A (KAT2A) jointly drive histone lactylation, thereby activating oncogenic pathways such as Wnt/β‐catenin and nuclear factor kappa‐B (NF‐κB) to enhance brain tumor growth and immune evasion.^[^ ^8^ ^]^ Beyond epigenomic modifications, key epigenetic regulators, non‐coding RNAs, and RNA modifications such as N6‐methyladenosine (m^6^A) modulate the differentiation and function of various immune cells in the TME, including T cells, natural killer (NK) cells, and macrophages.^[^ ^9^ ^]^ For example, inhibition of methyltransferase‐like 3 (METTL3) enhances T cell cytotoxicity and reverses T cell exhaustion by upregulating IFN‐γ and granzyme B, suggesting METTL3 as an epigenetic immune checkpoint.^[^ ^10^ ^]^ In summary, epigenetic modifications dynamically regulate tumor malignancy through multifaceted mechanisms, including transcriptional reprogramming of oncogenic networks, modulation of immune cell differentiation and function, and metabolic‐epigenetic crosstalk. These alterations collectively remodel the TME, thereby either promoting or suppressing tumor proliferation, metastasis, and immune evasion.

Notably, extensive crosstalk exists between epigenomic and epitranscriptomic modifications, potentially regulating the initiation and progression of diseases. The bidirectional crosstalk of chemical modification information flow between chromatin and RNA modifications substantially enhances the complexity of biological regulatory networks.^[^ ^11^ ^]^ A recent study demonstrated that the directional link between m^6^A and epigenomic modifications plays a critical role in complex human diseases.^[^ ^12^ ^]^ In various cancer types, the crosstalk between RNA modifications and epigenomic modifications occurs through two distinct modes: a “cis‐mode” mediating local effects when co‐transcription occurs and a “trans‐mode” inducing widespread downstream consequences.^[^ ^13^ ^]^ For instance, methyl‐CpG binding domain protein 6 (MBD6) can recognize and bind RNA 5‐methylcytosine (m^5^C), subsequently mediates H2A lysine 119 deubiquitination, and activates leukemia‐associated gene expression.^[^ ^14^ ^]^ Histone lactylation enhances METTL3 expression in tumor‐infiltrating myeloid cells, which increases m^6^A‐dependent JAK1 translation, thereby activating the JAK1‐STAT3 pathway to boost immunosuppressive functions.^[^ ^15^ ^]^ Investigating the crosstalk between epigenomic and epitranscriptomic modifications provides novel insights into the sophisticated epigenetic regulation underlying tumorigenesis. Their synergistic interactions establish a multi‐layered oncogenic regulatory network that collectively drives tumor malignancy. Combinatorial therapeutic strategies targeting this interactive network may pioneer new avenues for cancer treatment.

## 3 Tumor Immunometabolism Interacts with Epigenetic Modifications to Regulate Antitumor Immunity

**Figure 1:**
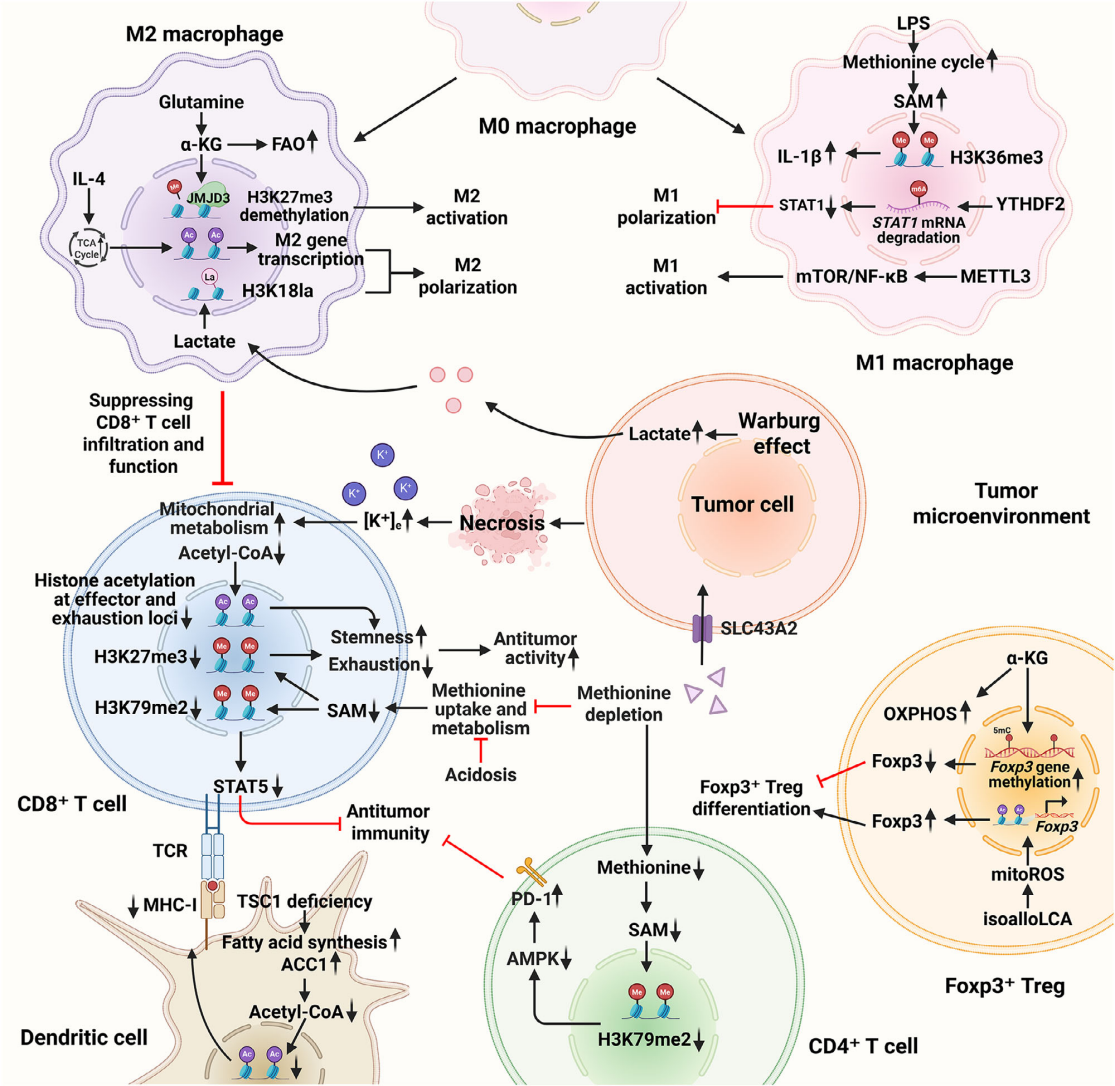
The role of epigenetic modifications in tumor immunometabolism. (Abbreviations: ACC1, acetyl‐CoA carboxylase‐1; α‐KG, α‐ketoglutarate; AMPK, AMP‐activated protein kinase; FAO, fatty acid oxidation; Foxp3, forkhead box protein P3; JMJD3, Jumonji domain‐containing protein 3; H3K18la, histone 3 lysine 18 lactylation; H3K27me3, histone 3 lysine 27 trimethylation; H3K36me3, histone 3 lysine 36 trimethylation; H3K79me2, histone 3 lysine 79 dimethylation; IL‐1β, interleukin‐1β; IL‐4, interleukin‐4; isoalloLCA, isoallolithocholic acid; [K⁺]_e_, extracellular potassium; LPS, lipopolysaccharide; METTL3, methyltransferase‐like 3; MHC‐I, major histocompatibility complex class I; mitoROS, mitochondrial reactive oxygen species; mTOR, mammalian target of rapamycin; NF‐κB, nuclear factor kappa‐B; OXPHOS, oxidative phosphorylation; PD‐1, programmed death 1; SAM, S‐adenosylmethionine; SLC43A2, solute carrier family 43 member 2; STAT1, signal transducer and activator of transcription 1; TCA, tricarboxylic acid cycle; TCR, T cell receptor; TSC1, tuberous sclerosis complex gene 1; YTHDF2, YTH N6‐methyladenosine RNA binding protein F2).

The epigenetic plasticity and metabolic reprogramming of immune cells are core mechanisms that regulate their phenotypes and functions. Key metabolites such as acetyl‐CoA and S‐adenosylmethionine (SAM) serve as essential substrates or cofactors for epigenetic modifying enzymes, dynamically modulating epigenetic processes to precisely control immune cell activation, differentiation, and effector functions (**Figure** **1**). This metabolic‐epigenetic regulatory network is intricately linked to immune dysregulation and tumor immune microenvironment remodeling, warranting deeper mechanistic dissection and translational exploration.

Metabolism‐mediated epigenetic regulation plays a crucial role in macrophage activation and polarization within the tumor microenvironment. For instance, interleukin‐4‐induced tricarboxylic acid cycle activation and tumor‐derived lactate promote M2 polarization through histone acetylation and lactylation, respectively. S‐adenosylmethionine, as a key methyl donor, participates in various epigenetic processes, including histone methylation to facilitate pro‐inflammatory macrophage phenotypes. Methionine deprivation and acidosis impair methionine uptake and metabolism in T cells, reducing S‐adenosylmethionine levels and histone methylation. This can not only decrease immune‐related gene expression to weaken antitumor effects but also enhance T cell stemness and limit exhaustion to potentiate antitumor immunity. Elevated extracellular potassium concentrations enhance T cell stemness by downregulating histone acetylation at effector and exhaustion‐related gene loci. In tuberous sclerosis complex 1‐deficient dendritic cells, enhanced acetyl‐CoA carboxylase 1‐dependent lipid metabolism depletes acetyl‐CoA and reduces histone acetylation, consequently impairing CD8^+^ T cell activation. Additionally, α‐ketoglutarate promotes oxidative phosphorylation and Foxp3 gene methylation in regulatory T cells, suppressing Foxp3^+^ regulatory T cell differentiation, whereas isoallolithocholic acid inhibits Foxp3^+^ regulatory T cell differentiation by increasing mitochondrial reactive oxygen species production.

### 3.1 Regulating the Activation and Differentiation of Immune Cells

Innate immune cells exhibit functional duality in the TME, mediating both antitumor immunity and tumor‐induced immunosuppression. These divergent outcomes can be orchestrated through coupled metabolic‐epigenetic control of their activation states. Tuberous sclerosis complex gene 1 (TSC1)‐deficient dendritic cells exhibit enhanced fatty acid synthesis, which exerts dual effects. Lipid accumulation impairs antigen cross‐presentation, while cytosolic acetyl‐CoA depletion reduces histone acetylation, subsequently downregulating major histocompatibility complex class I (MHC‐I) and IL‐7 expression. This metabolic‐epigenetic dysregulation ultimately compromises CD8⁺ T cell activation.^[^ ^16^ ^]^ Glutaminolysis‐derived α‐ketoglutarate (α‐KG) drives M2 macrophage activation and regulates lipid metabolic reprogramming in M2 macrophages through Jumonji domain‐containing protein 3‐mediated H3K27 demethylation.^[^ ^17^ ^]^

The metabolic–epigenetic regulatory axis also shapes immune cell differentiation, most notably influencing macrophages within the TME. Lipopolysaccharide‐stimulated macrophages upregulate the methionine cycle, elevating SAM levels and sustaining a high SAM:S‐adenosine homocysteine (SAH) ratio. This promotes a pro‐inflammatory macrophage phenotype by enhancing H3K36me3‐dependent inflammatory gene expression (e.g., IL‐1β).^[^ ^18^ ^]^ IL‐4 induces M2 polarization in the TME, accompanied by enhanced lactate metabolism and elevated tricarboxylic acid cycle activity. These metabolic shifts promote histone acetylation‐dependent M2 gene expression and reinforce immunosuppressive function.^[^ ^19^ ^]^ Moreover, tumor‐derived lactate can be transported into macrophages, inducing histone 3 lysine 18 lactylation (H3K18la) to drive M2 polarization. These M2 macrophages suppress CD8⁺ T cell infiltration and function, fostering an immunosuppressive TME that resists programmed death 1 (PD‐1) immunotherapy.^[^ ^20^ ^]^ RNA modifications also play crucial regulatory roles in macrophage activation and polarization. Specifically, METTL3 promotes M1 activation through the mammalian target of rapamycin (mTOR)/NF‐κB signaling pathway, while YTH N6‐methyladenosine RNA binding protein F2 (YTHDF2) negatively regulates M1 polarization by degrading m^6^A‐modified *STAT1* mRNA, thereby inhibiting glycolysis‐related gene expression.^[^ ^21^ ^]^

Adaptive immune cells are pivotal components of the tumor immune microenvironment, where their differentiation and activity determine the direction of immune responses, intricately orchestrated by the interplay between metabolic reprogramming and epigenetic modifications. Cell‐permeable α‐KG significantly enhances oxidative phosphorylation activity and promotes forkhead box protein P3 (Foxp3) gene methylation, inhibiting Foxp3^+^ Treg differentiation.^[^ ^22^ ^]^ The bile acid metabolite isoallolithocholic acid promotes Treg cell differentiation by enhancing mitochondrial reactive oxygen species (ROS) production and H3K27 acetylation at the Foxp3 promoter region.^[^ ^23^ ^]^ Moreover, Von Hippel‐Lindau deficiency has been reported to enhance the activity of glycolytic enzyme glyceraldehyde‐3‐phosphate dehydrogenase, which upregulates METTL3/METTL14 to promote m^6^A modification of inducible synergistic co‐stimulation molecules (ICOS) mRNA and suppress its expression. This mechanism ultimately leads to the suppression of follicular helper T cell development and differentiation.^[^ ^24^ ^]^

In summary, both innate and adaptive immune cells within the TME undergo profound metabolic reprogramming or are modulated by extracellular factors, leading to altered metabolite levels. These changes subsequently regulate immune cell activation and differentiation through epigenetic modifications. Current studies have predominantly focused on macrophage polarization, while studies on other immune cell types remain limited and warrant further investigation.

### 3.2 Modulating the Effector Function and Exhaustion of Immune Cells

When innate immune cells are initially exposed to pathogens or endogenous stimuli, they acquire enhanced responsiveness to secondary challenges through epigenetic modifications (e.g., histone methylation) and metabolic reprogramming (e.g., glycolysis, glutamine metabolism). This phenomenon is termed “trained immunity”.^[^ ^25^ ^]^ A recent study demonstrated that influenza‐trained, mucosal‐resident alveolar macrophages mediate durable, tissue‐specific, T cell–independent antitumor innate immunity. This process is linked to epigenetic, transcriptional, and metabolic resistance to tumor‐induced immunosuppression,^[^ ^26^ ^]^ although the latent mechanisms require further investigation. Moreover, in bladder cancer, lactate generated through aerobic glycolysis engages in a feed‐forward loop with M2 tumor‐associated macrophages (TAMs), driving the upregulation of METTL3. This enhances m^6^A modification of *programmed death‐ligand 1* mRNA, ultimately facilitating immune evasion and tumor progression.^[^ ^27^ ^]^

Current research on immunometabolism‐epigenetic modifications crosstalk primarily focuses on its regulatory effects in T cells. Tumor cells outcompete CD8^+^ T cells for methionine, impairing methionine metabolism and reducing intracellular SAM in T cells. This leads to loss of H3K79 dimethylation (H3K79me2) and decreased STAT5 expression, compromising antitumor immunity.^[^ ^28^ ^]^ Methionine deprivation also reduces H3K79me2 levels in CD4^+^ T cells, which downregulates AMP‐activated protein kinase activity, increases PD‐1 expression, and ultimately impairs antitumor immunity.^[^ ^29^ ^]^ Elevated extracellular potassium in the TME restricts nutrient uptake in CD8⁺ T cells, promoting mitochondrial metabolism while depleting acetyl‐CoA. This reduces histone acetylation and downregulates effector and exhaustion‐related genes, resulting in the formation of CD8⁺ T cells with stemness characteristics and enhanced antitumor function.^[^ ^30^ ^]^ Moreover, YTHDF1 upregulates monocarboxylate transporter 1 (MCT1) expression by enhancing its m^6^A modification, promoting lactate accumulation, and thereby impairing cytotoxic CD8⁺ T cell activity.^[^ ^31^ ^]^

Chronic antigen stimulation induces T cell exhaustion, characterized by diminished proliferative capacity, elevated inhibitory receptor expression, metabolic insufficiency, epigenetic alterations, and dysfunction. The acidic tumor microenvironment impairs methionine uptake and metabolism in T cells by downregulating methionine transporters, which alters H3K27me3 deposition at stemness‐associated gene promoters. This epigenetic reprogramming promotes the acquisition and maintenance of T cell stemness while limiting exhaustion.^[^ ^32^ ^]^ Recent findings demonstrate that ACSS2–P300 and ATP‐citrate lyase (ACLY)–KAT2A complexes orchestrate epigenetic remodeling during T cell exhaustion, highlighting metabolism–epigenetics coupling as a promising avenue for T cell–based antitumor therapies.^[^ ^33^ ^]^ Current evidence indicates that RNA modifications contribute to T cell exhaustion in tumors,^[^ ^10^ ^]^ yet whether this process involves metabolic regulation remains unclear.

Thus, tumor cell‐driven nutrient deprivation and the acidic TME collectively shape a distinct immunometabolic landscape. Current research has primarily elucidated how the metabolism‐epigenetics axis regulates T cell function, particularly metabolic competition‐induced epigenetic dysregulation driving T cell exhaustion or stem‐like properties. Notably, while metabolic and epigenetic alterations in immune cells may attenuate antitumor responses, they could also be leveraged to improve immune cell fitness and functionality. However, research in this area remains limited, especially regarding non‐T immune cells and RNA modifications. Furthermore, given the emerging paradigm of crosstalk between epigenomic and RNA modifications in reshaping tumor epigenetics, deeper investigations into their interplay with immunometabolic pathways are warranted.

## 4 Emerging Technologies for Elucidating the Crosstalk Between Epigenetic Modifications and Immunometabolism

**Figure 2:**
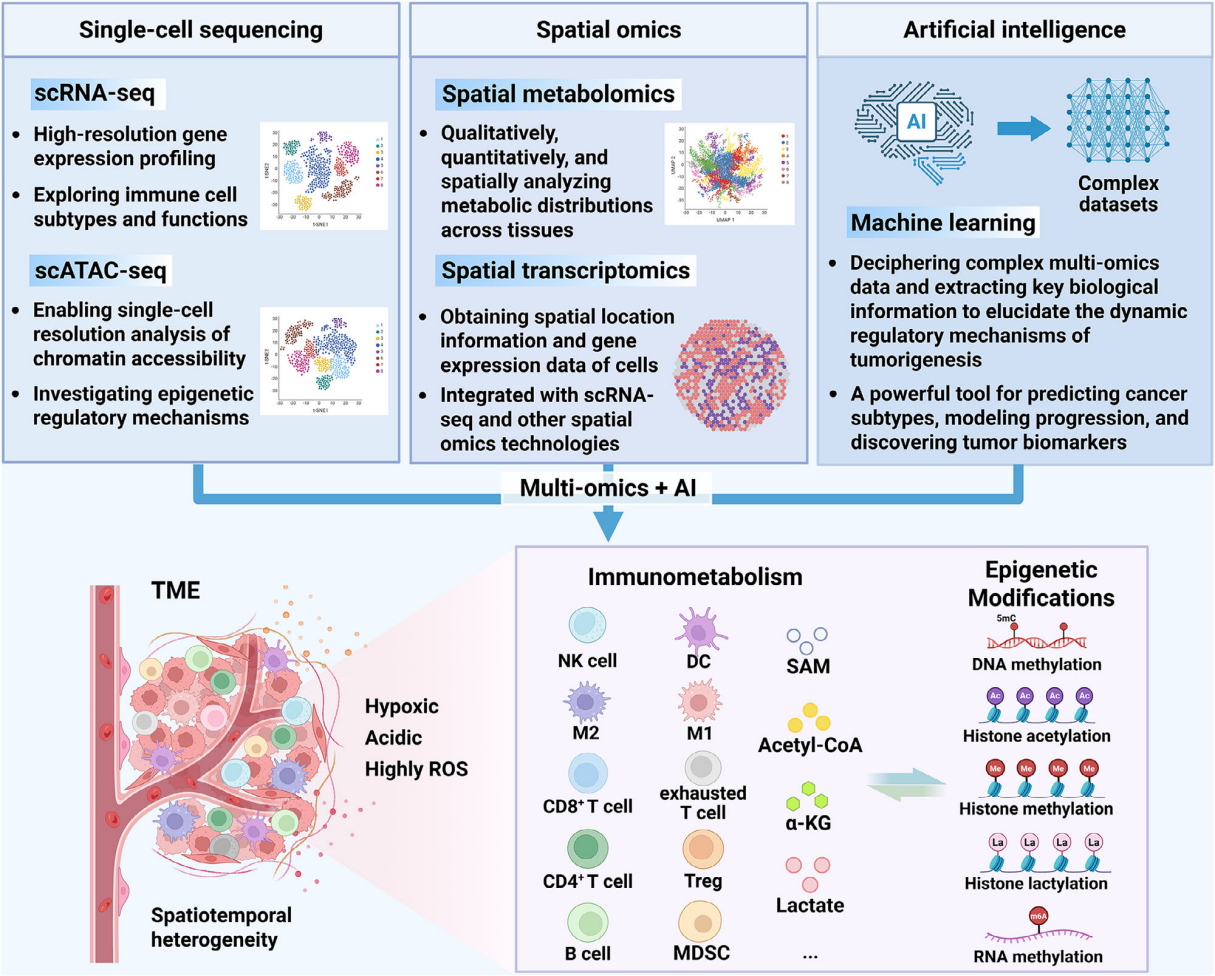
Novel technologies reveal epigenetic modification‐immunometabolic crosstalk in cancer. (Abbreviations: 5mC, 5‐methylcytosine; m^6^A, N6‐methyladenosine; α‐KG, α‐ketoglutarate; AI, artificial intelligence; DC, dendritic cell; MDSC, myeloid‐derived suppressor cell; NK cell, natural killer cell; ROS, reactive oxygen species; SAM, S‐adenosylmethionine; scATAC‐seq, single‐cell assay for transposase‐accessible chromatin using sequencing; scRNA‐seq, single‐cell RNA sequencing; TME, tumor microenvironment; Treg, regulatory T cell).

While research on the crosstalk between tumor immunometabolism and epigenomic modifications remains in its infancy, its translational significance is becoming increasingly evident. Heterogeneous tumor cell subpopulations, diverse infiltrating immune cells (e.g., myeloid cells, dendritic cells, and lymphocytes), and non‐cellular components collectively constitute the TME. These cellular elements exhibit uneven spatial distribution and undergo dynamic alterations during tumor progression, resulting in both spatial and temporal heterogeneity. This complexity presents significant challenges for deciphering the tumor immune microenvironment. While established techniques, including metabolomics, extracellular flux analysis, and clustered regularly interspaced short palindromic repeats (CRISPR) screening, have been widely employed in immunometabolism research, their inherent limitations remain non‐negligible.^[^ ^34^ ^]^ Most of these techniques target bulk cell populations and are disconnected from the native tissue microenvironment, limiting their ability to resolve inter‐subpopulation heterogeneity and capture spatial information.^[^ ^34^ ^]^ Promisingly, cutting‐edge single‐cell sequencing, spatial omics, and artificial intelligence are providing innovative avenues to advance the development of this field (**Figure** **2**).

The tumor microenvironment is a dynamic ecosystem comprising tumor cells, immune cells, stromal components, and extracellular factors. Its spatiotemporal heterogeneity plays a pivotal role in tumor initiation, progression, and metastasis. Metabolic reprogramming and intercellular communication collaboratively shape a tumor microenvironment characterized by hypoxia, acidosis, and accumulated reactive oxygen species. Notably, the crosstalk between immunometabolism and epigenetic modifications profoundly influences the activation, differentiation, and functional properties of immune cells. Emerging single‐cell sequencing technologies, spatial omics, and artificial intelligence empower the exploration of this field. Different technologies possess distinct advantages and characteristics, and their integrated application achieves complementary functionality. Thus, the integration of multi‐omics technology and artificial intelligence will provide a broader avenue for investigating the interplay between tumor immunometabolism and epigenetic modifications.

### 4.1 Single‐Cell Sequencing

Single‐cell sequencing technology overcomes the limitations of conventional next‐generation sequencing in analyzing cell‐specific information within highly heterogeneous cellular populations through single‐cell isolation, screening, and population identification at the single‐cell resolution. This approach has been extensively applied in tumor heterogeneity and tumor immune microenvironment research.^[^ ^35^ ^]^ Among them, single‐cell RNA sequencing (scRNA‐seq) and single‐cell assay for transposase‐accessible chromatin using sequencing (scATAC‐seq) have become widely used techniques for investigating immunometabolism and epigenetic regulation. The application of single‐cell sequencing technology has extended to the fields of epigenomics, proteomics, and metabolomics.^[^ ^35^ ^]^ Moreover, the integration of multi‐omics approaches provides a more comprehensive perspective for investigating the crosstalk between tumor immunometabolism and epigenetic regulation.

scRNA‐seq enables high‐resolution gene expression profiling of numerous individual cells simultaneously, allowing comprehensive assessment of diverse cell types, functional states, and subpopulations within heterogeneous cell populations. This powerful approach facilitates the identification of rare and novel cell subtypes, while providing unique insights into intercellular communication within the TME and the evolutionary dynamics of tumorigenesis.^[^ ^36^ ^]^ A recent study employing scRNA‐seq analysis demonstrated that the immunometabolic checkpoint cholesterol‐25‐hydroxylase is highly expressed in immunosuppressive TAM subsets and shows significant negative correlation with patient survival rates across multiple cancer types.^[^ ^37^ ^]^ In SET domain‐containing 2 (SETD2)‐deficient pancreatic tumors, scRNA‐seq identified a lipid‐enriched subpopulation of cancer‐associated fibroblasts (CAFs) and revealed critical connections between CAF heterogeneity, tumor cell epigenomic dysregulation, and their metabolic crosstalk.^[^ ^38^ ^]^

scATAC‐seq serves as a powerful tool for deciphering dynamic chromatin landscapes and gene expression regulation by sequencing DNA fragments from accessible chromatin regions, enabling single‐cell resolution analysis of chromatin accessibility to delineate transcriptional and epigenetic alterations.^[^ ^39^ ^]^ scATAC‐seq analysis uncovers the dynamic chromatin accessibility landscape of chimeric antigen receptor T cells (CAR‐T cells) under tumor stimulation, identifying basic leucine zipper transcription factor (BATF) and interferon regulatory factor 4 (IRF4) as key transcriptional regulators of CAR‐T cell exhaustion and elucidating the epigenetic regulatory mechanisms underlying CAR‐T cell dysfunction.^[^ ^40^ ^]^

Intriguingly, the integrated application of scATAC‐seq and scRNA‐seq enables simultaneous profiling of gene expression and chromatin accessibility in single cells. This multi‐omics approach facilitates direct linkage between regulatory elements and their target genes, identification of key transcription factors driving cell state transitions, and ultimately the construction of a more comprehensive gene regulatory atlas. The application of longitudinal scRNA‐seq and scATAC‐seq analyses enables the investigation of distinct transcriptional and epigenetic changes underlying T cell developmental trajectories, as well as the degree of heterogeneity within diverse T cell populations.^[^ ^41^ ^]^ Although both approaches provide advantages in single‐cell data acquisition, their analyses are highly susceptible to tumor heterogeneity and data noise, thereby limiting accuracy and reproducibility.^[^ ^36^ ^]^ Moreover, the loss of critical spatial context underscores the necessity of integration with other technologies such as spatial omics.

### 4.2 Spatial Omics

The advent of spatial omics has revolutionized cancer research paradigms by providing unprecedented insights into the spatial architecture and molecular characteristics of distinct cellular populations within tumors. This groundbreaking advancement enables the construction of comprehensive “spatial maps” of malignancies, facilitating in‐depth exploration of the intricate spatial relationships and cellular interactions within the TME, thereby offering novel insights into tumor heterogeneity and progression mechanisms.^[^ ^42^ ^]^

Spatial metabolomics integrates mass spectrometry imaging with metabolomics to qualitatively, quantitatively, and spatially analyze metabolic distributions and heterogeneity across tissues. This approach is increasingly utilized to uncover cancer‐associated metabolic reprogramming and identify potential biomarkers.^[^ ^43^ ^]^ Therefore, spatial metabolomics provides a powerful tool for deciphering the spatial distribution of metabolic activities in immune cells within the TME. Spatial transcriptomics based on sequencing and imaging methods enables systematic measurement of spatial expression profiles for all or most genes in tissues, deciphering precise characterization of intratumoral heterogeneity and crucial molecular features at tumor‐normal tissue interfaces.^[^ ^44^ ^]^

Despite limitations in resolution, sensitivity, and reproducibility, ongoing technological innovations and the integration of multi‐omics approaches are progressively overcoming these challenges.^[^ ^43^, ^44^ ^]^ Currently, spatial transcriptomics is increasingly integrated with scRNA‐seq and other spatial omics (e.g., spatial metabolomics) for investigating tumor immune microenvironment and metabolic reprogramming. Integrated spatial transcriptomics and scRNA‐seq across multiple cancer types identified four distinct CAF subtypes with heterogeneous spatial distribution and metabolic reprogramming features, wherein inflammatory CAFs orchestrate immunosuppressive microenvironment formation through modulating immune cell interaction networks.^[^ ^45^ ^]^ By integrating single‐cell transcriptomics, spatial transcriptomics, and spatial metabolomics, researchers visualized the spatial co‐localization of metabolites and gene expression in pancreatic cancer. This analysis identified characteristic metabolites and uncovered aberrant interactions between cancer cells and macrophages, thereby providing mechanistic insights and potential therapeutic avenues for targeting tumor metabolism.^[^ ^46^ ^]^ A study integrated spatial transcriptomics and spatial metabolomics to characterize the spatial distribution and interaction networks of immune cells in the oral squamous cell carcinoma microenvironment, while elucidating the pivotal role of polyamine metabolism dysregulation in promoting tumorigenesis and immune evasion.^[^ ^47^ ^]^

While current research has yet to fully utilize these technologies to explore the interplay between tumor immunometabolism and epigenetic regulation, single‐cell sequencing and spatial omics offer remarkable potential for this field. Through delineating the region‐specific distribution of metabolites and the spatial expression patterns of epigenetic modifiers and their target genes, the integration of single‐cell sequencing and spatial omics promises to elucidate how immunometabolic reprogramming governs local immune responses through epigenetic mechanisms in tumors.

### 4.3 Artificial Intelligence

The development of machine learning models significantly enhances the capability to extract critical biological information from complex cancer datasets, providing robust support for cancer subtype prediction, disease progression modeling, tumor biomarker discovery, and early clinical diagnosis.^[^ ^43^ ^]^ Machine learning approaches elucidated the regulatory roles of immune‐metabolism‐related genes (IMRGs) in immune infiltration and tumor stemness in neuroblastoma, and subsequently established an IMRG‐based prognostic prediction model.^[^ ^48^ ^]^ By integrating spatial transcriptomics and scRNA‐seq data, machine learning algorithms decipher multi‐omics data, elucidating the dynamic regulatory mechanisms in tumorigenesis while establishing clinically translatable scoring systems for precise diagnosis and prognostic stratification.^[^ ^49^ ^]^ When integrated with spatial transcriptomics, machine learning algorithms can predict gene expression from histopathological images, enhancing the interpretability of tissue architecture and cellular interactions. This synergy holds promise for advancing clinical decision‐making by refining prognostic assessment and guiding therapeutic strategies.^[^ ^44^ ^]^ By integrating high‐dimensional data, machine learning enables the deciphering of complicated interactions among single‐cell multi‐omics, spatial omics, and metabolomics datasets, thereby precisely identifying critical regulatory hubs in tumor immunometabolism and epigenetic regulation. Although the application of artificial intelligence in studying the crosstalk between tumor immunometabolism and epigenetic regulation remains nascent, its integration with multi‐omics technologies demonstrates remarkable potential, offering a novel methodological paradigm for decoding this intricate regulatory network.

## 5 Concluding Remarks

Tumor immunometabolism and epigenetic modifications are interconnected through a sophisticated regulatory network, wherein their bidirectional crosstalk reshapes the TME through multiple mechanisms. Tumor cells alter immune cell metabolism by competing for nutrients and secreting metabolic byproducts, thereby functionally reprogramming immune responses. Metabolic intermediates generated during immune cell metabolic reprogramming serve as epigenetic modulators that regulate the expression of immune‐related genes, ultimately controlling immune cell activation, differentiation, and functional states. Concurrently, alterations in epigenetic modifier activity may reciprocally influence metabolic pathways. For instance, lactate‐induced histone lactylation modulates m^6^A modification by altering the expression of m6A‐regulating enzymes, while m^6^A modification reciprocally regulates lactate levels and histone lactylation by controlling lactate transporters and glycolytic pathways.^[^ ^20^, ^31^ ^]^ The immunometabolic‐epigenetic crosstalk plays a pivotal role in tumor immune evasion, driving immunosuppressive TAM polarization and T cell exhaustion. Deciphering this regulatory network not only advances our understanding of tumor immune tolerance mechanisms but also provides a conceptual framework for developing novel therapies targeting the metabolism‐epigenetics axis.

Nevertheless, several critical questions remain unresolved. Currently, most studies remain focused on correlative observations, failing to elucidate how specific metabolic pathways directly drive epigenetic alterations in immune cells or identify the key signaling pathways mediating this crosstalk to regulate immune cell activity and function. Resolving these questions necessitates precise experimental designs and dynamic monitoring to establish definitive mechanistic causal links. In addition, future investigations should broaden their scope to encompass more non‐T immune cell populations, as well as RNA modifications. Given the growing recognition of the crosstalk between epigenomic and epitranscriptomic modifications in tumors, their coordinated regulatory mechanisms and functional roles in tumor immunometabolism hold substantial scientific value and warrant further investigation. Owing to the pronounced heterogeneity of tumors, the immunometabolism–epigenetic regulatory axis may vary significantly across tumor types and even among subtypes of the same tumor. This complicates systematic elucidation of its underlying mechanisms, yet also suggests that specific immunometabolic products or epigenetic regulators may hold potential as biomarkers for tumor diagnosis. Moreover, targeting the metabolic–epigenetic axis has emerged as a promising therapeutic strategy, whereas the potential off‐target effects and resistance associated with epigenetic drugs remain major challenges.^[^ ^50^ ^]^ Encouragingly, combinatorial approaches that integrate metabolic or epigenetic agents with immunotherapies have demonstrated notable synergistic effects,^[^ ^51^ ^]^ yet rigorous clinical trials are still required to establish their efficacy and safety.

In recent years, multi‐omics integration has emerged as a cornerstone approach for deciphering TME complexity. The synergistic application of single‐cell sequencing and spatial omics has driven groundbreaking advances in tumor immunometabolism and epigenetic regulation. Single‐cell technologies resolve transcriptional profiles and chromatin accessibility at single‐cell resolution, whereas spatial omics preserve crucial spatial context, enabling comprehensive characterization of metabolic heterogeneity and immune cell spatial interactions. Through multi‐omics integration, researchers have identified immune cell subsets with distinct metabolic‐epigenetic features and elucidated their metabolic reprogramming mechanisms and immunoregulatory functions within the TME. This high‐resolution, multi‐dimensional paradigm provides a transformative framework for investigating the immunometabolic‐epigenetic crosstalk in cancer. Furthermore, artificial intelligence demonstrates unique capabilities in decoding the interplay between tumor immunometabolism and epigenetic regulation by integrating high‐dimensional multi‐omics data and uncovering pivotal biological mechanisms within complex datasets.

## Conflict of Interest

The authors declare no conflict of interest.

## Author Contributions

X. X., W. L., and P. Z. contributed equally to this work. W.M. and W.L. conceived this project. X.X., W.L., and P.Z. wrote the original draft and designed the figures. W.M., B.T., and P.L. revised the manuscript. W.M., B.T., and P.L. supervised this project. W.M. got financial support. All authors reviewed and approved the final manuscript.

## Peer Review

The peer review history for this article is available in the Supporting Information for this article.

## Supporting information

Supplementary Information: Record of Transparent Peer Review

## Data Availability

Data sharing not applicable to this article, as no datasets were generated or analysed during the current study.
