# Supplementary material for: Delving Into the Dialogue between Epigenetic Modifications and Immunometabolism in Cancer
Source: Adv Genet (Hoboken). 2025 Sep 5;6(3):e00034. doi: 10.1002/ggn2.202500034 (PMC12482933; doi:10.1002/ggn2.202500034)
Supplement: Supplementary file 1 — Supplementary Information: Record of Transparent Peer Review [file GGN2-6-e00034-s001.docx]

Record of Transparent Peer Review

Delving into the Dialogue Between Epigenetic Modifications and Immunometabolism in Cancer

Data submitted: 28-May-2025

1st Editorial Decision: 27-July-2025

1^st^ Revision Received: 23-Aug-2025

2^nd^ Editorial Decision: 25-Aug-2025

Last Revision Received: 27-Aug-2025

Accepted: 27-Aug-2025

Revision with responses: 23-Aug-2025 (1^st^ Revision )

**Reviewer 1:**

This perspective focuses on the interplay between immune metabolism and epigenetic modifications in cancer, highlighting their functional implications. This manuscript is concise and well-organized, systematically introducing major epigenetic regulatory mechanisms. It further illustrates their roles in immune cell activation, differentiation, functions, and exhaustion through representative examples. Additionally, the authors discuss and project the utility of single-cell sequencing, spatial omics, and artificial intelligence in advancing this research area. The accompanying cartoon visually integrates metabolic reprogramming in diverse tumor-infiltrating immune cells, epigenetic alterations, and their crosstalk with cancer cells, forming a comprehensive framework—commendable. Nevertheless, several critical points require further clarification, as outlined below:

**Major Comments:**

**1.Comments to the Author:**

Page 6, line 9: The author only provided one example for "cis-mode" and "trans-mode", but it is unclear whether this example belongs to the cis category or the trans category. And what about another example?

**Author response and action taken**:

Thank you for your profound question. In the original manuscript, the example we provided belongs to the category of a "trans-mode." As per your suggestion, we have added a "cis-mode" example and arranged both examples in the order mentioned in the preceding sentence. The detailed content (**Page 6, Line 132~134)** in our perspective is as follows.

**2.Comments to the Author:**

Figure 1:"H3K27 acetylation" at the Foxp3 promoter region" has not been accurately depicted in Figure 1.

**Author response and action taken**:

Really thanks for your reminder. We have corrected the panel in Figure 1(**Page 7, Line 156)** regarding "H3K27 acetylation at the Foxp3 promoter region" as follows.

**3.Comments to the Author:**

The abbreviations and full names used in the text are not consistent. Sometimes, only the full name is provided without the abbreviation.

Thank you for your advice. We have carefully reviewed the manuscript and refined the use of abbreviations throughout. For some terms that appear only once in the text, we have provided the full form instead of abbreviating them. All abbreviations have been standardized according to your suggested guidelines.

**Minor Comments:**

**1.Comments to the Author:**

Page 5, lines 31-34: acetyl-CoA synthetase 2 and lysine acetyltransferase 2A ---- acetyl-CoA synthetase 2 (ACSS2) and lysine acetyltransferase 2A (KAT2A).

**Author response and action taken**:

Thank you for your advice. We have revised the manuscript according to your suggestions as follows (**Page 5, Line 110~111)**.

**2.Comments to the Author:**

Page 5, line 40/Page 6, line 1: m6A ---- m^6^A (6 should be placed in the upper right corner).

**Author response and action taken**:

Really thanks for your reminder. We have carefully reviewed the relevant expressions throughout the manuscript and made consistent revisions to ensure clarity and uniformity. The detailed content (**Page 5, Line115; Page 5, Line 128)** in our perspective is as follows.

**3.Comments to the Author:**

Page 7，Figure 1 legends: "+" should be placed in the upper right corner.

Page 8, line 27: Tuberous sclerosis complex gene 1 ---- Tuberous sclerosis complex gene 1 (TSC1).

**Author response and action taken**:

Really thanks for your reminder. We have revised the manuscript according to your suggestions as follows (**Page 7, Line 169~171; Page 8, Line 188~189)**.

**4.Comments to the Author:**

Page 8, line 46: SAM:S-adenosine homocysteine ratio ---- SAM:S-adenosylhomocysteine (SAH) ratio.

**Author response and action taken**:

Thank you for your advice. We have revised the manuscript according to your suggestions as follows **(Page 8, Line 199~120)**.

**5.Comments to the Author:**

Page 9, line 9: STAT1 mRNA ---- *STAT1* (italic) mRNA. The name of the gene should be in italics.

**Author response and action taken**:

Thank you for your advice. We have revised the manuscript according to your suggestions as follows **(Page 9, Line 212)**.

**6.Comments to the Author:**

Page 13, line 50/56: ScATAC-seq

**Author response and action taken**:

Thank you for your advice. We have revised "ScATAC" in the original manuscript to "scATAC" as follows **(Page 14, Line 346)**.

**Reviewer 2:**

This perspective article reviews the relationship between epigenetic regulation and immunometabolism within the tumor microenvironment. The authors summarize how metabolic shifts in immune cells are connected to epigenetic changes, such as DNA methylation and histone modifications. The manuscript is timely, well-written, and addresses a subject of high interest for cancer biology and immunology.

**Major Points**

**1.Comments to the Author:**

While the manuscript does an excellent job of summarizing the current literature, it would be more impactful as a "perspective" piece if it offered a clearer vision for the future of the field. The authors should consider adding a discussion of what they view as the most critical unanswered questions and the next conceptual hurdles. For instance, a key challenge is moving beyond observing correlations to establishing a causal link between specific metabolic pathways and epigenetic outcomes in immune cells.

**Author response and action taken**:

We sincerely thank you for your valuable comments. In the fifth section of the article, we have added a new paragraph to summarize the key challenges and unresolved issues in investigating tumor epigenetic modifications and immunometabolism. The detailed content **(Page 17, Line 444~Line 454)** in our perspective is as follows.

**2.Comments to the Author:**

The discussion on RNA modifications is less developed than the sections on DNA and histone modifications. To provide a more balanced overview, the authors should expand on this topic. It would be beneficial to include more examples of how RNA-modifying enzymes are controlled by the metabolic state of a cell and how these modifications subsequently impact immune cell function.

**Author response and action taken**:

We appreciate your thoughtful comments. We have complemented the discussion on the role of RNA modifications (**Page 5, Line 116~119; Page 10, Line 241~245; Page 10, Line 256~258**) in tumor immunity. Despite an extensive literature search, studies specifically focusing on metabolite-driven RNA modifications affecting immune cell function remain limited. Additionally, we have expanded our discussion on the link between RNA modifications and tumor immunometabolism (**Page 16~17, Line 435~438)**, providing a more precise and detailed explanation to better highlight their close connection and pivotal role in tumor progression. The detailed content in our perspective is as follows.

**3.Comments to the Author:**

The section on therapeutic implications is promising but could be explored in greater depth. The authors should elaborate on the challenges and opportunities associated with targeting the metabolism-epigenetics axis for cancer therapy. This could include a discussion of potential off-target effects, the development of drug resistance, and the exciting possibilities of combination therapies that pair metabolic or epigenetic drugs with immunotherapy.

**Author response and action taken**:

We are grateful to you for your constructive suggestions. We fully agree that investigating the therapeutic significance of the crosstalk between tumor epigenetic modifications and immunometabolism is of great importance. Based on your suggestion, we have revised the manuscript by adding a dedicated discussion (**Page 17, Line 450~454)** in the fifth section, where we elaborate on the challenges and opportunities of targeting the metabolic–epigenetic axis as a potential strategy for cancer therapy. The detailed content in our perspective is as follows.

**Minor Points**

**1.Comments to the Author:**

In Table 1, some abbreviations in the "Reader" column (e.g., "YEATS," "HBO1") are not defined in the legend. Please ensure all terms are clearly explained.

**Author response and action taken**:

Thank you for your advice. We have carefully reviewed the abbreviations (**Page 4, Line 92~99)** in Table 1. Unfortunately, full names of “YEATS” and “HBO1” are rarely mentioned in the literature. We have therefore provided an alternative widely recognized name for “HBO1” based on its description in the article "The histone lysine acetyltransferase HBO1 (KAT7) regulates hematopoietic stem cell quiescence and self-renewal" published in Blood in February 2022 (DOI: 10.1182/blood.2021013954). The detailed content in our perspective is as follows.

**2.Comments to the Author:**

The manuscript is well-written, but some sentences are quite long and complex. Revising these for brevity and clarity would improve overall readability.

**Author response and action taken**:

Thank you for your advice. We have polished the language throughout the text, including removing redundant expressions and breaking down long, complex sentences to improve readability. Below are a few specific examples (**Page 8, Line 201~204; Page 10, Line 237~241)** of the modifications we have made.

**3.Comments to the Author:**

The reference list is thorough. To ensure the perspective is as current as possible, consider including a few key studies that have been published in late 2024 or early 2025.

**Author response and action taken**:

Thank you for your advice. We have updated several references, replacing older literature with newer, high-quality articles to enrich the content and incorporate cutting-edge findings. Below are a few newly cited high-quality articles published in the last two years.

[1] Wu K, Li S, Hong G et al. Targeting METTL3 as a checkpoint to enhance T cells for tumour immunotherapy. Clin Transl Med 14(11), e70089 (2024).

[2] Zou Z, Dou X, Li Y et al. RNA m5C oxidation by TET2 regulates chromatin state and leukaemogenesis. Nature 634(8035), 986–994 (2024).

[3] Ma S, Dahabieh MS, Mann TH et al. Nutrient-driven histone code determines exhausted CD8+ T cell fates. Science 387(6734), eadj3020 (2025).

[4] Nie J, Wang C, Zheng L et al. Epigenetic agents plus anti-PD-1 reprogram the tumor microenvironment and restore antitumor efficacy in Hodgkin lymphoma. Blood 144(18), 1936–1950 (2024).

**Reviewer 3:**

This manuscript explores the crosstalk between tumor immunometabolism and epigenetic modifications, offering potential new insights into cancer biology. The topic is timely and aligns with growing interest in the metabolic and epigenetic regulation in tumor immunity. However, several critical issues must be addressed to ensure the academic rigor and depth of perspective. Therefore, this manuscript needs revision that are suggested as follows:

**1.Comments to the Author:**

While the perspective centers on the crosstalk between tumor epigenetic modifications and immunometabolism, the discussion appears selectively focused. For instance, epigenomic alterations are relatively well covered, whereas RNA modifications receive limited attention. Expanding this aspect would help achieve a more balanced and comprehensive overview.

**Author response and action taken**:

We sincerely thank you for your valuable comments. We have complemented the discussion on the role of RNA modifications in tumor immunity (**Page 5, Line 116~119; Page 10, Line 241~245; Page 10, Line 256~258**). Compared with extensive studies on DNA and histone modifications, investigations specifically addressing metabolite-driven RNA modifications in regulating immune cell function remain limited, yet equally important. Accordingly, we have further emphasized the necessity of advancing research in this area in the discussion (**Page 16~17, Line 435~438; Page 17, Line 446~449**). The detailed content in our perspective is as follows.

**2.Comments to the Author:**

The discussion on the interplay between epigenomic and RNA modifications in disease pathogenesis (Point 2) is insightful. To reinforce the conceptual framework, the authors are encouraged to further elaborate in Point 3 on how this crosstalk may intersect with tumor immunometabolism—particularly how RNA modifications (e.g., m⁶A) may coordinate with DNA or histone modifications to regulate oncogenic immunometabolic reprogramming.

**Author response and action taken**:

Really thanks for your reminder. We fully agree that the interplay between epigenomic and RNA modifications represents an important and noteworthy topic in tumor pathogenesis. However, after extensive literature review, we were only able to identify studies reporting the role of such crosstalk in regulating immune cell functions within tumors (**Page 8~9, Line 204~208**), while direct evidence linking it to oncogenic immunometabolic reprogramming remains scarce. Thus, we emphasized in the discussion (**Page 17, Line 447~449**) the importance of further investigations in this direction. The detailed content in our perspective is as follows.

**3.Comments to the Author:**

On page 11, the authors briefly mention the limitations of established methods like metabolomics in immunometabolism research. Clarifying these limitations and contrasting them with the strengths of emerging technologies would improve the manuscript's analytical depth.

**Author response and action taken**:

We sincerely thank you for this insightful suggestion. In the revised manuscript, we have explicitly highlighted the limitations of conventional approaches (**Page 11~12, Line 294~296**) relative to single-cell technologies and spatial omics, thereby clarifying their comparative limitations. The detailed content in our perspective is as follows.

**4.Comments to the Author:**

This perspective would be strengthened by incorporating more recent high-impact studies (particularly from the past two years) to better reflect cutting-edge developments in the field. A more detailed analysis of emerging investigative approaches (e.g., spatial multi-omics, artificial intelligence) would offer more forward-looking insights that are expected in a perspective article.

**Author response and action taken**:

Thank you for your valuable comments. We have updated several references, replacing older literature with newer, high-quality articles to enrich the content and incorporate cutting-edge findings. The detailed content (**Page 15, Line 388~393; Page 16, Line 415~418**) in our perspective is as follows.

**5.Comments to the Author:**

The writing should be more concise and logically connected. Some passages are unnecessarily wordy, and clearer transitions between ideas would help readers follow the argument. Certain paragraphs resemble fragmented literature summaries rather than cohesive arguments, especially the second paragraph of the second point, "Common epigenetic modifications that modulate tumor immunity." Careful editing to tighten phrasing and improve flow would enhance readability.

**Author response and action taken**:

Thank you for your advice. We have thoroughly polished the language throughout the manuscript and improved the logical flow, particularly in the second paragraph of the second point. The detailed content (**Page 5, Line 100~123)** in our perspective is as follows.

**Reviewer 4:**

In this perspective by Xie et al., titled “Delving into the Dialogue Between Epigenetic Modifications and Immunometabolism in Tumor”, the authors offer a timely and conceptually engaging discussion on the intersection of epigenetic regulation and immunometabolism in cancer progression. The topic is highly relevant, and the conceptual framework is intriguing. Below are a few suggestions for improvement:

**1.Comments to the Author:**

The manuscript explores the interplay between epigenetic modifications and immunometabolism in cancer; however, much of the current discussion centers on how metabolic intermediates shape epigenetic regulation. It would be valuable to further explore how epigenetic modifications, in turn, affect immunometabolism.

**Author response and action taken**:

Thank you for your profound question. While studies investigating the regulatory effects of epigenetic modifications on immunometabolism remain relatively limited, we fully agree that this aspect is equally important. Accordingly, we have added relevant content (**Page 16~17, Line 435~438)** to the article, as follows.

**2.Comments to the Author:**

Please take a moment to double-check the classification of epigenetic “writers,” “readers,” and “erasers” in Table 1, to ensure accuracy in the listed molecules and their functional annotations.

**Author response and action taken**:

Thank you very much for the reminder. We have carefully reviewed the contents of Table 1 to ensure accuracy and consistency. All abbreviations used in the table (**Page 4)** have been clearly defined.

**3.Comments to the Author:**

The fourth section highlights several emerging technologies that may facilitate the study of interactions between epigenetic modifications and immunometabolism. However, it would be beneficial to briefly acknowledge their current limitations to provide a more balanced perspective.

**Author response and action taken**:

Thank you for your kind advice. In response, we have complemented the limitations of emerging technologies, thereby underscoring both the necessity for continuous technological innovation and the importance of integrating multi-omics approaches. The detailed content (**Page 14, Line 358~362; Page 15, Line 380~382)** in our perspective is as follows.

**4.Comments to the Author:**

The writing would benefit from some polishing to enhance clarity and scientific tone. This includes adjusting informal phrasing, defining abbreviations consistently upon first mention (e.g., “acetyl-CoA” and “NK cells” in the second paragraph of the second point), and ensuring terminologies are precise and context-appropriate throughout.

**Author response and action taken**:

We have polished the language throughout the manuscript and standardized the abbreviations as your suggestion. The specific revisions (**Page 5, Line 110~111; Page 5, Line 116)** are outlined below.

2^nd^ Revision with responses: 27-Aug-2025

This version of the manuscript features a more visible title, a strengthened abstract, and expanded concluding remarks.
